# Epigenomics of Neural Cells: REST-Induced Down- and Upregulation of Gene Expression in a Two-Clone PC12 Cell Model

**DOI:** 10.1155/2015/202914

**Published:** 2015-08-27

**Authors:** Jose M. Garcia-Manteiga, Silvia Bonfiglio, Maria Luisa Malosio, Dejan Lazarevic, Elia Stupka, Davide Cittaro, Jacopo Meldolesi

**Affiliations:** ^1^Scientific Institute San Raffaele, Center for Translational Genomics and Bioinformatics, 20132 Milan, Italy; ^2^CNR Institute of Neuroscience, 20129 Milan, Italy; ^3^Humanitas Research Center, Rozzano, 20089 Milan, Italy; ^4^Boehringer Ingelheim, 88400 Biberach an der Riß, Germany; ^5^Division of Neurosciences, San Raffaele Institute and Vita-Salute San Raffaele University, 20133 Milan, Italy

## Abstract

Cell epigenomics depends on the marks released by transcription factors operating via the assembly of complexes that induce focal changes of DNA and histone structure. Among these factors is REST, a repressor that, via its strong decrease, governs both neuronal and neural cell differentiation and specificity. REST operation on thousands of possible genes can occur directly or via indirect mechanisms including repression of other factors. In previous studies of gene down- and upregulation, processes had been only partially investigated in neural cells. PC12 are well-known neural cells sharing properties with neurons. In the widely used PC12 populations, low-REST cells coexist with few, spontaneous high-REST PC12 cells. High- and low-REST PC12 clones were employed to investigate the role and the mechanisms of the repressor action. Among 15,500 expressed genes we identified 1,770 target and nontarget, REST-dependent genes. Functionally, these genes were found to operate in many pathways, from synaptic function to extracellular matrix. Mechanistically, downregulated genes were predominantly repressed directly by REST; upregulated genes were mostly governed indirectly. Among other factors, Polycomb complexes cooperated with REST for downregulation, and Smad3 and Myod1 participated in upregulation. In conclusion, we have highlighted that PC12 clones are a useful model to investigate REST, opening opportunities to development of epigenomic investigation.

## 1. Introduction

REST (RE-1 silencing transcription factor, otherwise called NRSF), a repressor of gene expression, is known to play different roles in neural and nonneural cells. In neural cells, due to the increased proteasomal degradation initiated during differentiation, the levels of REST are low. As a consequence, REST repression is also low, the expression of possible target genes is increased, and their role in the specific structure and function of these cells is relevant [1, 2]. In contrast, in most nonneural cells levels of REST are high, and repression of target genes, induced by REST binding to RE-1, a DNA sequence included in the promoters and other regulatory regions, is also high. Possible REST targets were initially believed to be over 2,000 [3–9]. Recently, however, upon integrated computational analyses of available REST ChIP-Seq datasets, the number of these genes was increased to almost 6,000 [10–15].

For many years, the study of REST repression was carried out mostly in nonneural cells. The investigation of REST targets in neural cells was limited [16–19]. Such investigation has been recently extended to neuronal populations differentiated* in vitro* from human pluripotent stem cells (iPSCs) [20]. Such populations, however, include neuronal subtypes at various stages of maturation together with neuronal progenitors and glial cells that express high levels of REST. Therefore, the data on differentiated iPSCs should be considered with caution [20].

In the present study, the role of REST has been investigated by the comparison of two clones of PC12, a mature neural cell line isolated from a rat pheochromocytoma [21], often employed as a neuronal model [22, 23]. Wild-type PC12 (wtPC12) cells, characterized by very low REST [22, 23], do not account for the properties of the whole line. In fact, a few PC12 clones spontaneously exhibit a peculiar phenotype [24–26] and REST levels 50–80-fold higher than wtPC12 [27, 28]. Transfection of wtPC12 clones with the repressor and of high-REST PC12 (hrPC12) clones with dominant negative constructs was shown to attenuate the differential properties of the two types of clones, suggesting many differences between the two clones to be due to their different REST levels [28–30]. Therefore, an epigenetic investigation of REST does not require the parallel use of neural and nonneural cell lines, or the manipulation of one such line, but can be done by comparing the properties of appropriate clones of the same PC12 line.

A few thousand gene transcriptome analyses of hrPC12 and wtPC12 clones, carried out in 2002 by a first generation microarray technology [31], had remained without functional integration with sequence analyses, such as the ChIP-Seq ENCODE and databases, made available only recently. This limitation prevented epigenetic interpretations. Repression by REST, occurring mostly via the deacetylation and methylation/demethylation of histone H3 [1, 2, 10, 15, 32], is known in fact to induce generation of highly informative marks of the epigenetic events taking place in the corresponding genes [32].

By the RNA-Seq investigation of gene expression in hrPC12 and wtPC12 clones we have now reached a global transcriptome landscape: ~15,500 genes, with ~8,000 gene ontology annotations of function, 1,770 of these genes strongly down- and upregulated in the hrPC12. Our RNA-Seq studies have not been complemented with ChIP-Seq analyses. However, by the integration with the available ChIP-Seq datasets and the Roadmap Epigenomics project we have identified, among the up- and downregulated genes, those possibly governed directly by the repressor and those that appear governed indirectly, via the involvement of other factors, in particular transcription factors (TFs) [1, 2]. The transcription signature analysis has revealed a cooperation of REST with the Polycomb repressor complexes (PRCs) [33–36]. The synergism REST/PRC, which operates in the control of many genes and several neural phenotype pathways, appears to play relevant roles in the epigenomic regulation. Two other TFs, Smad3 [37] and Myod1 [38], were found to be involved in the upregulation. In conclusion, only a fraction of the down- and upregulated genes of the RNA-Seq list, the possible direct targets of REST for ChIP-Seq and Roadmap Epigenomic databases, might be of special interest for the epigenetic evaluation.

## 2. Materials and Methods

### 2.1. RNA Sequencing and Data Analysis

The RNA, extracted with the RNeasy Mini Kit (Qiagen, Valencia, CA) from two, carefully washed clones of PC12, the wtPC12 and the hrPC12 previously referred to as PC12–27 [24, 25, 28, 30], was analyzed with the Agilent 2100 Bioanalyzer (Agilent Technologies, Santa Clara, CA). Libraries, prepared starting from 2 *μ*g of RNA/sample with the Illumina TruSeq RNA Sample Prep kit v2 procedure, were quantified by the Qubit BR assay (Life Technologies, Illkirch, France) and the Agilent 2100 Bioanalyzer and sequenced on the Illumina HiSeq 2000 platform. Quality control of the obtained reads and alignment to the rat reference genome (RGSC3.4/rn4) were performed using FASTQC suite with default parameters (FastQC, a quality control tool for high throughput sequence data, http://www.bioinformatics.babraham.ac.uk/projects/fastqc/) and the TopHat aligner [39]. Gene expression read counts were exported and analyzed in R to identify differential expressed genes (DEGs), using the* DESeq* Bioconductor library [40]. Genes with a baseMean value < 5 for all samples, or showing 0 reads as baseMean in either wtPC12 or hrP12, were filtered out to avoid infinite and 0 values of log_2_ fold changes (FC). *p* values were adjusted using a threshold for false discovery rate (FDR) ≤ 0.01 using the Benjamini and Hochberg correction [41]. Genes listed as DEGs are shown in Table S1. Genes additionally filtered for an absolute value of log_2_ FC > 2 (total 1,770) were used for further analysis. Box-whiskers and Volcano plots analyses were performed using R standard functions. Variance stabilizing transformation function in* DESEq* library of expression values was plotted with the* heatmap* library after scaling to* Z*-score of the rows. Raw data are available through Gene Expression Omnibus (GEO (http://www.ncbi.nlm.nih.gov/geo/) with accession number GSE59946).

### 2.2. Enrichr Enrichment Analysis

Rat gene symbols were translated into their human orthologs using BioMart [42].* Enrichr* tool [43] was used programmatically through specific python scripts to query the different databases.

Adjustment for multiple comparisons [41] was applied (*p* adj. function in R) and results were filtered by using two cutoffs: adj. *p* value < 0.05 and* Z*-score < −1.5. The* Z*-score applies a correction to the Fisher exact test *p* value that* Enrichr* developed in order to correct for the possible false positive results coming from large gene sets giving lower *p* values with big gene overlaps.* Z*-scores less than −1.5 represent pathways performing better than random combinations of genes in terms of *p* value. The combined score (log (*p* value) *∗ Z*-score) serves to rank terms, taking into account both approaches [43].

### 2.3. TF ChIP-Seq: Pathways and Epigenomics Enrichment Analyses

ENCODE TF ChIP-seq and ChEA [44] databases were used for TF analyses. KEGG, BioCarta, WikiPathways, and Reactome were used for pathway analyses. TF protein-protein interactions (PPIs) were used for the upregulation signature. Epigenomic Roadmap project data was used to find enrichment in epigenomic signatures using sequencing technologies that map DNA methylation, histone modifications, chromatin accessibility, and small RNA transcripts (http://www.roadmapepigenomics.org/) [32].

### 2.4. Gene-Pathway Networks

*Enrichr* results were imported into the R environment, and genes in significant pathways were interconnected to build networks of genes to pathways via the igraph R library.

### 2.5. TF Targets and Histone Marks Definition

Among the fractions of the hrPC12 DE genes, REST potential targets were defined as those found to overlap significantly with enriched ChIP-Seq datasets, thus immunoprecipitated by the anti-REST antibody in at least one cell type and context, gene overlapping our RNA-Seq experimental cell model. Similarly, the PRC potential targets were defined as subunits found to overlap significantly with enriched ChIP-seq datasets of the subunits Suz12, Ehz2, Rnf2, or Jarid2. Histone marks in Roadmap Epigenomics datasets were considered if overlapped significantly with our RNA-Seq data.

### 2.6. Statistical Tests

Median log_2_ fold changes were compared using a two-sample Wilcoxon/Mann-Whitney test (wilcoxon.test function in R). *p* values < 0.05 were considered significant.

## 3. Results

### 3.1. Global RNA-Seq Signature

**Figure 1:**
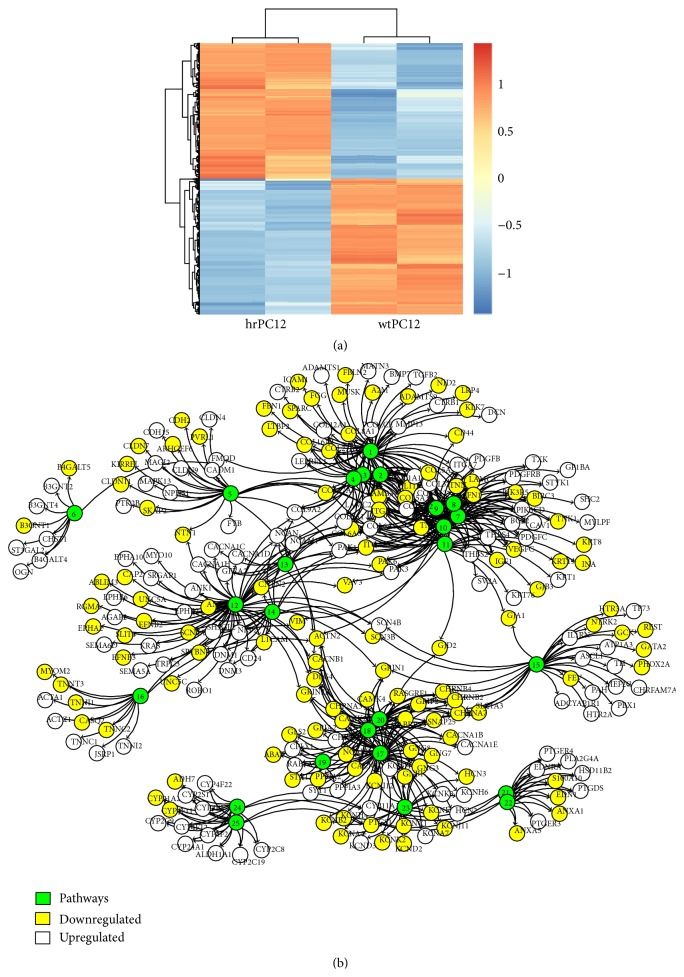
Differential expression of genes down- and upregulated in hrPC12 with respect to wtPC12 cells. Functional gene pathways. (a) shows the Heatmap comparative expression of the 1,770 DE genes analyzed in two samples of the hrPC12 (left) and wtPC12 (right). Normalized log_2_ values were used (after variance stabilizing transformation) and scaled. The almost 900 upregulated genes are distributed on the top and the almost 900 downregulated genes on the bottom. (b) illustrates a series of pathways (green circles). The afferent downregulated genes are in the yellow circles and the upregulated genes in the white circles. The numbers that appear in green circles mark groups of genes identified as follows: 1–4: extracellular matrix; 5: cell-cell signaling; 6: keratan sulfate biosynthesis; 7–11: focal adhesion, cell communication; 12–14: axon guidance; 15: membrane channels and signaling by NGF and TF involved in neuron development; 16: contraction; 17–20: synaptic transmission; 21 and 22: prostaglandin biosynthesis; 23: excitability; 24 and 25: enzymes and metabolism.

In order to carry out the global RNA-Seq transcriptome landscape investigation, ~90 million reads, corresponding to 100 bp paired-end reads, were produced on an Illumina HiSeq 2000 platform for each PC12 clone, with high quality scores. The sequence reads mapping the rat genome were counted, and the differentially expressed (DE) gene values of the two clones were compared using the DESEq package in Bioconductor [40]. Adjusted (adj.) *p* value corrections for false discovery rate were calculated according to Benjamini and Hochberg [41]. Collectively, the comparative analysis in the two clones yielded a list of 1,770 DE genes with absolute log_2_ fold changes (hrPC12/wtPC12) (log_2_ FC) > 2 and adj. *p* values < 0.01 after multiple test corrections. After applying log_2_ FC and adj. *p* value cutoffs, almost 900 downregulated and almost 900 upregulated genes were identified (Figure 1(a); see Table S1 in Supplementary Material available online at http://dx.doi.org/10.1155/2015/202914). Eleven groups of phenotypic pathways were identified based on RNA-Seq data of both down- and upregulated genes (adj. *p* values < 0.05). Among these, the predominant pathways were 4, dealing with axon guidance, synapse transmission, focal adhesion, and extracellular matrix (Figure 1(b)).

### 3.2. ChIP-Seq Enrichment Analysis

In this section the RNA-Seq information about DE gene expression was integrated with available ChIP-Seq enrichment datasets. Recent studies had shown that the occupation by REST of the various chromatin regions and the ensuing changes of gene transcription occur differently in various contexts [20]. To reduce the risks dependent on differences among cell lines, the correlation with the hrPC12 DE genes revealed by RNA-Seq (Table S1) was established using ChIP-Seq enrichment within data available not from one, but from numerous distinct types of cells, all reported in the ENCODE TF datasets (https://www.encodeproject.org), and in a manually curated database (ChEA database), covering 212 TFs and their possible targets through 241 PubMed reported publications [44]. Analysis of the data was carried out through the* Enrichr* suite [43] by which the ChIP-Seq data present in the databases were employed to perform the gene set enrichment analyses. The results revealed, among the DE genes down- and upregulated in the hrPC12, 67 and 70 significantly enriched ENCODE TF and ChEA datasets, with adj. *p* values < 0.05 and* Z*-scores < −1.5 (Tables S2 and S3, resp.).

**Figure 2:**
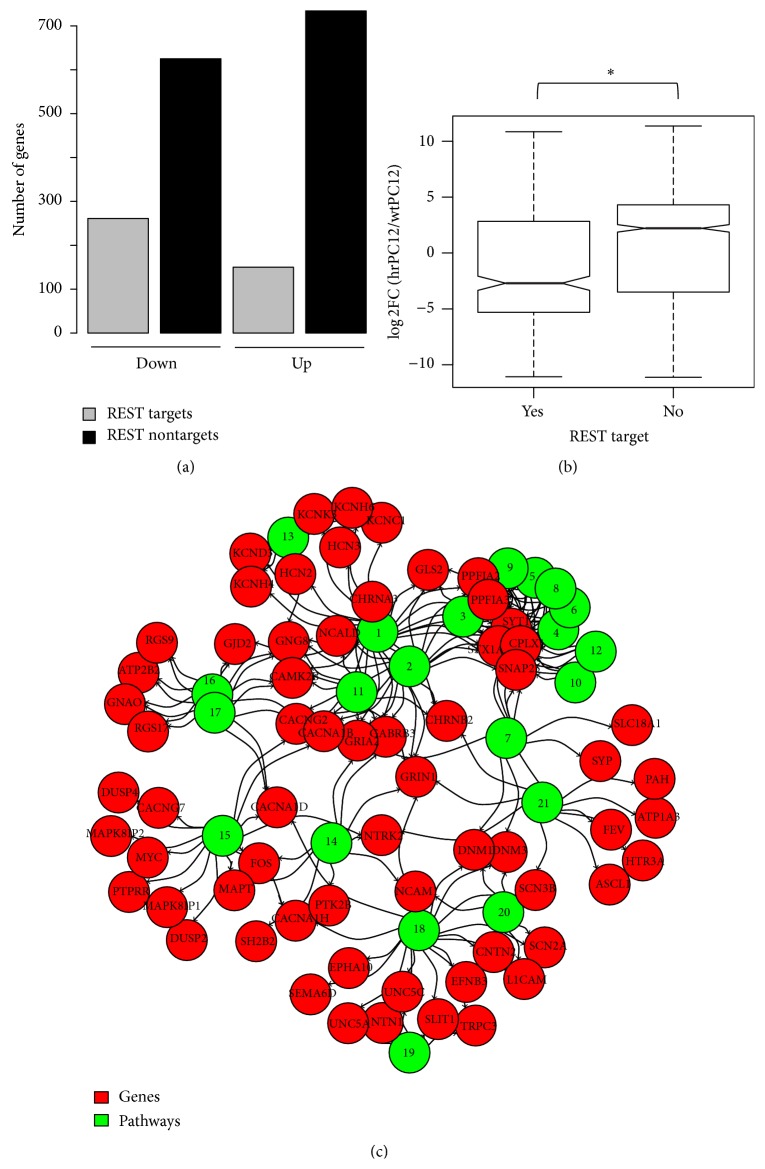
Enrichment analyses of hrPC12 signatures based on available ChIP-Seq datasets and ENCODE TF data derived from cell lines of various types. (a) Among the genes identified by RNA-Seq as down- and upregulated, the ChIP-Seq enrichment analyses distinguished two families, one composed of the genes possibly regulated directly by REST (REST targets) and the other regulated indirectly or by other mechanisms (non-REST targets). (b) The down- and upregulated genes analyzed together were separated as possible REST targets (YES) and nontargets (NO), illustrated as box-whiskers in terms of median fold distribution of the hrPC12/wtPC12 log_2_ ratios. Notice that the notches, corresponding to the median log_2_ ratios, are negative (−2.7) for the targets and positive (1.9) for the nontargets. (c) illustrates the phenotypic pathways of possible downregulated REST target genes exhibiting significant ChIP-Seq enrichment. The green circle numbers correspond to the following pathways: 1–12: synaptic transmission; 13: excitability: 14–17: cell-cell signaling; 18–20: axonal guidance; 21: SIDS syndrome, including neural-specific TFs and receptors.

The ChIP-Seq enrichment analyses revealed that 29.5% (261/886) of the hrPC12 genes downregulated according to RNA-Seq were potential direct REST targets, while 70.5% (625/886) were not (Figure 2(a)). The top 10 ChIP-Seq datasets enriched in downregulated genes exhibited REST in the first place [45] followed by a series of datasets concerning PRC subunits (Ezh2, Suz12, and Rnf2). In three additional ENCODE ChIP-Seq datasets, obtained from the HCT116 (human colon cancer), U-87 (human glioblastoma), and Panc-1 (pancreatic cancer) lines, the enrichment of REST was also highly significant (Table 1). In the upregulated genes of hrPC12, the potential REST targets were less numerous than among the downregulated genes (150/884 = 17.0%), while the REST nontargets were more numerous (734/884 = 83.0%) (Figure 2(a)). None of the datasets significantly enriched in these genes included REST. Among the top hits we found TFs abundant not in neural but in various biological contexts, such as muscle differentiation (Tcf12, Myod1) and cell development (Ep300, Tps3, and Smad3) (Table 2).

The downregulation of possible REST target and nontarget genes in hrPC12 cells relative to wtPC12 cells was then correlated with their level of expression documented by their median fold ratios, that is, of log_2_ FC (hrPC12/wtPC12) (Wilcoxon *p* value < 2.2 × 10^−16^). The possible REST target genes were found to exhibit a predominant downregulation (median fold ratio −2.7) while the possible nontarget genes exhibited a predominant upregulation (median fold 1.9) (Figure 2(b)).

The properties of the possible REST target/nontarget genes were then investigated in terms of their phenotypic pathway enrichments. Interestingly, among the downregulated genes, the possible REST nontarget genes failed to exhibit any significantly enriched pathway (adj. *p* value < 0.05 and* Z*-score < −1.5), whereas the downregulated, possible REST target genes exhibited high enrichment for neural phenotypic pathways (Figure 2(c); Table S4). A number of these pathways (synaptic transmission, excitability, cell-cell signaling, and axon guidance) coincided in part with those identified also among the whole genes identified by RNA-Seq (Figure 1(b)). It appears therefore that the downregulated gene signature in hrPC12 cells was dominated by possible REST target genes, rather than by possible REST nontarget genes.

**Figure 3:**
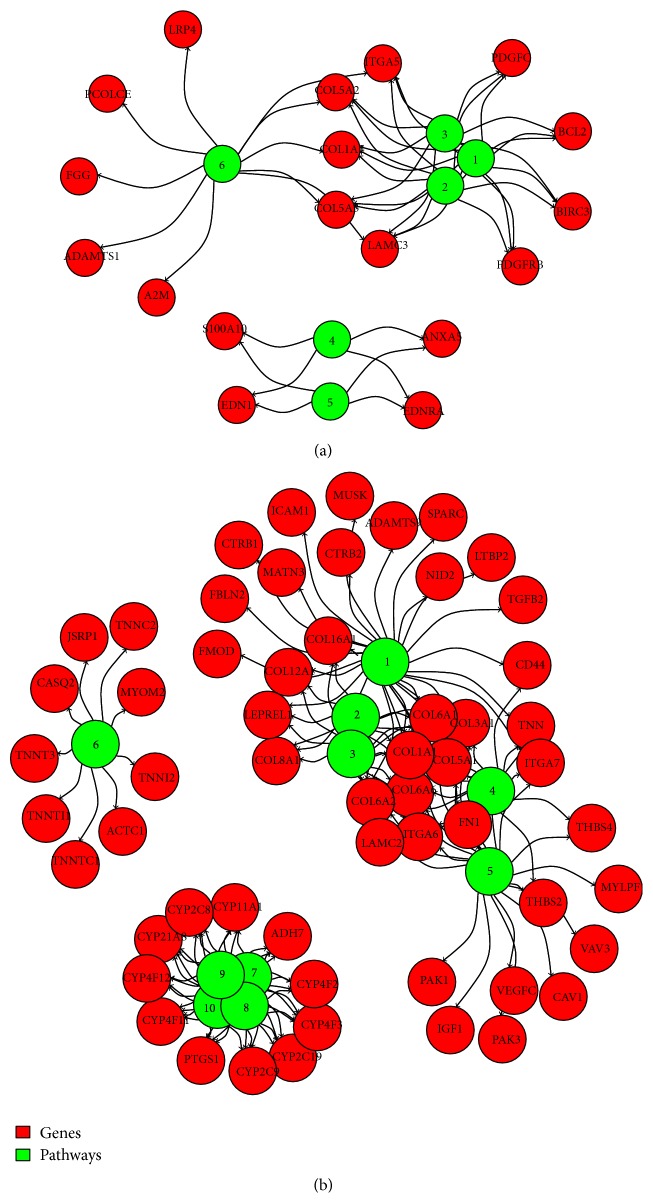
Phenotypic pathways of the ChIP-Seq enriched, upregulated genes of hrPC12 cells. (a) shows pathways of possible REST target genes: 1–3: focal adhesion; 4 and 5: PG synthesis; 6: extracellular matrix. (b) shows pathways of possible REST nontarget genes. 1–3: extracellular matrix organization. 4: receptor interaction; 5: focal adhesion; 6: muscle structure/function; 7–10: enzymes and metabolism.

The correlation between possible REST target/nontarget and their enrichment was investigated also in the upregulated genes. In this case the enrichment analyses (Table S5) showed a few phenotypic pathways related to both possible REST target and nontarget genes. Of the pathways of possible REST target genes, one was involved in focal adhesion and the other, containing several collagen genes, belonged to the extracellular matrix (adj. *p* value < 0.1;* Z*-score < −1.5) (Figure 3(a)). Among the possible REST nontarget genes the enrichment was mainly composed of nonneural pathways, including muscle contraction, extracellular matrix organization, and metabolism (Figure 3(b)).

### 3.3. Mechanisms of Gene Down- and Upregulation: Possible Dependence on REST, on the Cooperation of REST with PRC, on PRC Only, and on Other Mechanisms

**Figure 4:**
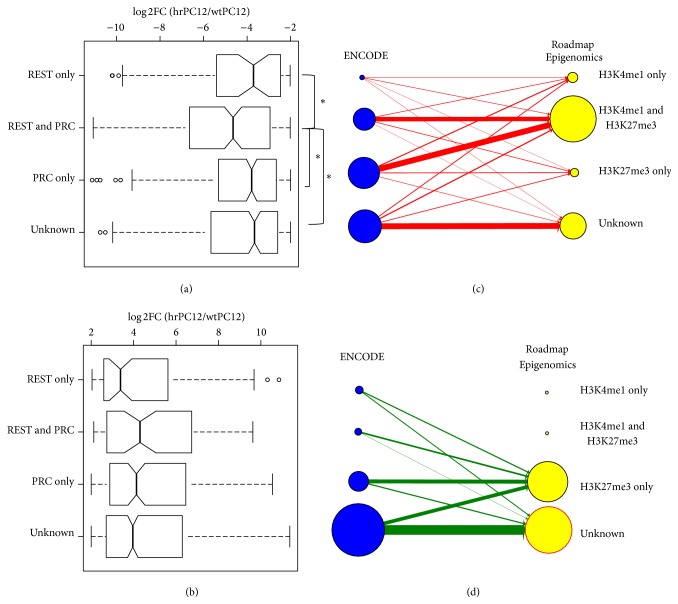
Possible mechanisms of gene down- and upregulation in hrPC12 cells analyzed by ChIP-Seq enrichment and Roadmap Epigenomics datasets. (a) and (b) show downregulated (a) and upregulated (b) genes of hrPC12 cells, separated in the four subfamilies dependent on their possible mechanism of regulation and illustrated as box-whiskers in terms of median fold distribution of the hrPC12/wtPC12 log_2_ ratios. Notice that the notches (median ratios) are approximately the same for the subfamilies of both the down- and upregulated genes except for the downregulated, possible REST and PRC regulated subfamily, whose median ratio is significantly lower than those of the other downregulated subfamilies, suggesting the combined repression by the REST and PRC repression systems. (c) and (d) illustrate the distribution in the four subfamilies of the same downregulated (c) and upregulated (d) genes as revealed by the ChIP-Seq (blue circles) and Roadmap Epigenomics (yellow circles) datasets. In the presentation of the results obtained by the two approaches, three gene subfamilies are named differently: the subfamily REST only in the ChIP-Seq is named H3K4me1 only in the Roadmap Epigenomics; REST and PRC are H3K4me1 and H3K27me3; PRC only is H3K27me3 only. Notice that the two target and the two nontarget subfamilies predominate among the downregulated and the upregulated genes, respectively. These differences appeared to be markedly reinforced in the Roadmap Epigenomics analyses. In the figure the differential gene distributions revealed by the two approaches are illustrated as gene redistributions of different relevance among the subfamilies, illustrated as arrows of various thicknesses (red in (c), green in (d)).

The distinction of both down- and upregulated genes in two families, the possible REST target and nontarget families, already defined in Figure 2(a), was reconsidered here upon separation of each family into two subfamilies, one enriched and the other not enriched significantly in either ChIP-Seq dataset of PRC subunits (Jarid2, Suz12, Ezh2, and Rnf2). The blue circles shown in Figure 4(c) illustrate the contributions of the four subfamilies of downregulated genes. Of the two possible REST target gene subfamilies, the one that appeared to be possibly downregulated by REST only accounted for ~5.1%; the one in which downregulation appeared possibly dependent on REST together with PRCs accounted for ~24.4%. Of the two potential REST nontarget subfamilies, the one enriched in the PRC subunits, however without REST, accounted for ~34.3%; the one showing no REST and no PRC, apparently dependent on still unknown mechanisms, accounted for ~36.2%.

The results of this section, relative to the genes illustrated by the blue circles, were analyzed also in terms of median fold, calculated from the RNA-Seq differential hrPC12/wtPC12 transcription data. The median fold values (log_2_ FC (hrPC12/wtPC12)) of the possible REST target genes downregulated by REST only and the values of the possible nontarget genes downregulated by PRC only and by unknown mechanisms were all very close (−3.7, −3.8, and −3.7 ratios). Only the possible target genes of the REST/PRC-dependent downregulation subfamily showed significantly lower median log_2_ FC values (median ratio −4.6) (Wilcoxon *p* value < 2.2 × 10^−16^) (Figure 4(a)). These results suggest that the combination of the REST/PRC repressor system increases the downregulation of gene expression compared to the levels reached by the repressor systems alone.

The results obtained by the same approach with the upregulated genes (blue circles of Figure 4(d)) were substantially different from those of downregulated genes. The number of genes apparently governed by REST only, although small (~9.2%), was higher, while the numbers governed by PRCs, with REST or alone (~8.0 and ~22.9%, resp.), were much lower than the corresponding numbers of downregulated genes. In contrast, the number of the genes from the possible REST nontarget subfamily governed by unknown mechanisms was higher (~60.0%) (Figure 4(b)). In terms of calculated RNA-Seq median fold ratios, the values for all upregulated subfamilies were similar, with median close to 4, except for the REST only target genes that exhibited a ratio only slightly and nonsignificantly lower than the others (Figure 4(b)).

The blue circle results shown in Figures 4(c) and 4(d) refer to the gene subfamilies enriched by the available ENCODE ChIP-Seq datasets. The same subfamilies could be reinvestigated using however the Roadmap Epigenomics databases program (http://www.roadmapepigenomics.org), making reference to two dynamic properties of the H3 histone tail sites related to the activity of REST and PRC: the monomethylated K4 (a substrate of LSD1, a demethylase component of the REST complex [1, 2]) and the trimethylated K27 (a substrate of the PRC2 complex [33, 34, 36]). An advantage of the two approaches is that in both cases the genes attributed to the various sub-subfamilies are known. The overlap genes in TF target and Epigenomics Histone Marks subfamilies can therefore be identified. The size of the connecting arrow between differently coloured circles provides an indication about the size of the overlap.

The results obtained by this second approach are illustrated by yellow circles in direct comparison to the Chip-Seq enrichment data of the blue circles shown in Figures 4(c) and 4(d). In the downregulation section (Figure 4(c)), the subfamily positive only for H3K4me1 was found to exhibit a significant larger number of genes (~11.3 versus 5.1%) with respect to the REST only of the ChIP-Seq enrichment subfamily (blue circle), due to the contribution of the other downregulated subfamilies. The H3K4me1/H3K27me3 subfamily was also enlarged (~51.1 versus 24.4%) with respect to the corresponding REST/PCR blue circle, reaching the largest value among the four downregulated subfamilies. Also in this case the enlargement was due to transfer of genes, especially from both the PRC only and the unknown subfamilies. In contrast, the yellow circles were clearly reduced compared to the corresponding blue circle subfamilies, very much the H3K27me3 only subfamily (~9.2 versus 34.3%) and less markedly the unknown subfamily (~29.5 versus 36.2%). In both these cases the changes were mostly due to the apparent gene transfer, especially to the H3K4me1/H3K27me3 subfamily.

Analogous Roadmap Epigenomics results were obtained with the upregulated subfamilies of Figure 4(d). The H3K4me1 and the H3K4me1/H3K27me3 subfamilies remained almost inappreciable compared to the corresponding blue circles of the ChIP-Seq enrichment results. A large accumulation was in the H3K27me3 subfamily that exhibited a ~46.4 versus 23.0%, an increase due to the apparent transfer from the H3K4me1/H3K27me3 and unknown subfamilies. The last, unknown subfamily, in contrast, was decreased however moderately (~53.6 versus 60.0%) with respect to its ChIP-Seq enrichment blue circle.

Taken together, the results obtained with the Roadmap Epigenomics approach reinforced the possible role of REST, mostly in cooperation with PRC, as the direct repressors of the downregulated genes. On the other hand, the direct contribution of the PRC only and the unknown mechanisms to downregulated genes appeared less relevant. Interestingly, the latter two subfamilies appeared to be the only ones in which many upregulated genes were recovered.

### 3.4. Network Analysis of Upregulated Signatures

**Figure 5:**
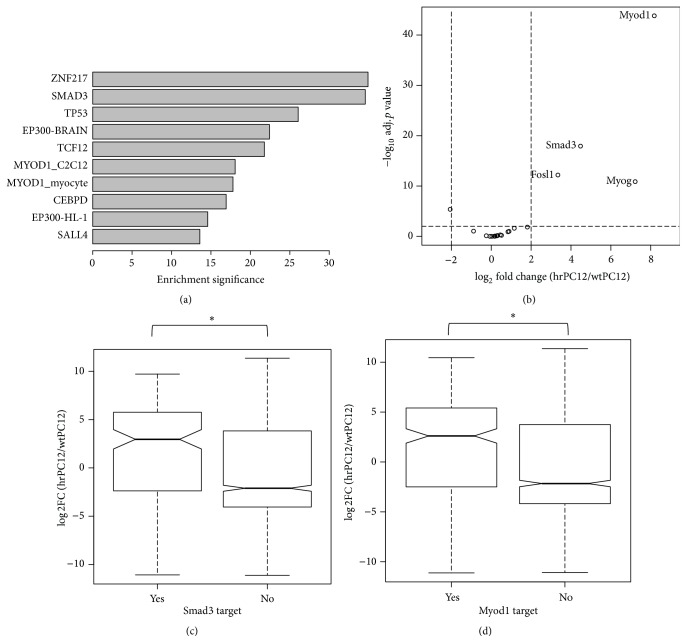
Network analysis of upregulated signatures. (a) The top 10 TFs enriched among the upregulated signatures expressed in our PC12 cells are listed based on their significance (−log_10_, adj. *p* value < 0.01). (b) Volcano plot of significant upregulated TFs enriched in our RNA-Seq datasets showing DE genes (i.e., log_2_ fold change and −log_10_, adj. *p* value < 0.01). (c) illustrates the median fold of hrPC12/wtPC12 log_2_ ratios concerning the potential Smad3 and Myod1 target (YES) and nontarget (NO) genes, illustrated as box-whiskers. The significantly higher notches document that the target genes of the two transcription factors are comparatively upregulated.

A list of TFs potentially involved in gene upregulation was generated, from the enriched ChEA and ENCODE datasets based on ChIP-Seq enrichment analyses (Table S3). The genes expressed at detectable levels in our RNA-Seq data (Figure 5(a)) were derived from the top TFs shown in Table 2. Moreover, in order to explore in detail the signature of the genes upregulated in hrPC12 cells, we employed a TF PPI network (Table S6) [43]. The PPI network can be used to calculate the enrichment of TF interactions in a gene list and thus to expand the information about the physical TF-DNA interactions. Among the analyzed TFs, only one, Smad3, visible already in the TF list of the ChIP-Seq data (Figure 5(a)), was found to reach a significant enrichment in the network (adj. *p* value < 0.01,* Z*-score < −1.9) (Table S6). We next investigated whether the upregulated expression of TFs was affecting the expression of their target genes. In the case of Smad3 and Myod1, two TFs significantly upregulated (together with Fosl1 and Myog) in the hrPC12 cells (Figure 5(b)), the median fold analysis revealed the expression of targets genes to be also significantly upregulated compared to nontarget genes (Wilcoxon *p* value < 2.2 × 10^−16^) (Figure 5(c)). This result suggests the possible involvement of Smad3 and Myod1 in the upregulated phenotype of hrPC12 cells.

## 4. Discussion

This work was carried out by using two appropriately selected PC12 clones. The combination of these two clones is an interesting model for the investigation of REST, a transcription repressor playing a master role in the development and the specificity of neurons and neural cells [27, 28, 30]. PC12 is a well-known, rat neuron-like cell line that includes spontaneously, together with the highly frequent low-REST wtPC12 clones, also a few hrPC12 clones with 50–80-fold higher levels of REST. A few previous studies demonstrated that both wtPC12 and hrPC12 acquire properties (gene expression, phenotype) of the other clone upon changes of their REST levels [27–30]. These results strongly suggest that many gene expression differences existing between wtPC12 and hrPC12 clones can be sustained by their differences in REST levels. Taking into account that the specific binding of REST to its gene targets is converted into gene repression via relevant histone marks, that is, the deacetylation and methylation/demethylation of the N terminal tail of adjacent H3 histones, the PC12 model appears to be of potential, great interest for the investigation of epigenetic problems.

In the present work, the experimental RNA-Seq investigation identified the genes that are down- and upregulated in the hrPC12 compared to wtPC12 clone cells. The work was expanded by its integration with data on genes function, revealed by two approaches, enrichment analyses of available Chip-Seq datasets of several predominantly human cell lines and occurrence of targets specific of REST and PRC complexes. These integrations have revealed new aspects of REST control of gene expression. Future expansions of these studies at the epigenetic level could be envisaged based not only on the comparison of the two clones but also on changes of the REST levels in either clone induced, for example, by stimulation, inhibition, and pharmacological treatments.

Based on our RNA-Seq results, we discovered that many DE genes, both down- and upregulated in the hrPC12 clone, are not random but participate in distinct phenotypic pathways. The combination with the enrichment analyses of ChIP-Seq datasets has revealed that the pathways are frequent in the possible REST target family of downregulated genes and rare in the nontarget family. Among the upregulated genes this difference, although not as evident, was still present. The identification of many pathways is a new finding. In the past, in fact, only two pathways were identified, including downregulated genes involved in neurosecretion and excitability [31].

The most important results, concerning both genes down- and upregulated and identified by RNA-Seq in hrPC12 cells, have been obtained by their combination upon translation into their human orthologue genes, with two functional approaches, the first being the enrichment analyses calculated from the available ChIP-Seq datasets. To avoid the risk to operate with ChIP-Seq from only a cell type different from PC12, we have employed datasets from numerous cell lines of both neural and nonneural types and considered their information only when the overlap with our RNA-Seq data was highly significant. The second approach, the analyses from the Roadmap Epigenomics datasets, was based on the finding of H3 histone sites related to the activity of REST and PRC.

The mechanisms expected to participate in the transcription signature of the DE genes were numerous: direct effects, right of REST, working alone or in collaboration with PRCs, another repressor system operative via epigenetic processes [33–36]; indirect effects dependent on the REST repression/activation of PRCs or other genes such as TFs. The latter possibility appears quite likely because in the list of DE genes of hrPC12 (Table S1) TFs are quite numerous. Finally we cannot exclude that some down- and/or upregulations are induced independently of the action of REST, for example, to compensate effects induced in hrPC12 cells by the latter repressor.

The two approaches employed here have revealed that the DE genes of down- and upregulation could be both distributed in four subfamilies, characterized by at least partially different mechanism of repression/stimulation. Interestingly, however, the relevance of the subfamilies revealed by the two approaches was profoundly different. Specifically, with the enrichment ChIP-Seq approach, the predominance appeared for the indirect mechanisms in both cases, accounting for the 70.5% and 83.0% of the down- and upregulated genes. In contrast, with the Roadmap Epigenomics approach the indirect mechanisms were predominant only for the upregulated genes (almost 100%), whereas for the downregulated the predominance was for the REST-governed, direct mechanisms, including REST only (11.3%) and REST together with PRC (51.0%). The latter REST/PRC combination subfamily appeared particularly efficient in downregulation because it was the only one of this work showing a median fold ratio lower than those of the other subfamilies.

The differential predominance of transcription signatures that emerges from the second approach appears to make sense. Direct REST repression, that in our results was found to account for over 62.0% of the downregulated genes, is well known [1, 2], and its cooperation with PRC has been reported repeatedly [33, 34, 36]. The possibility that the remaining 38.0% downregulated genes are repressed indirectly, for example, via REST repression of an activating TF, Smad or by independent mechanisms, appears quite likely. Even more convincing is the almost complete absence of direct REST among the mechanisms of gene upregulation. Although REST has been previously reported to work also as a stimulator of transcription [46, 47], this effect is likely to be indirect, mediated by the repression of either repressor TFs or other factors. In the present work we have explored the possible role of TFs in gene upregulation. Two of these TFs, Smad3 and Myod1 [37, 38], which are upregulated activators of transcription, were found to stimulate the transcription of their specific target genes. The role of these two and of other TFs and their possible dependence on REST remain however to be further investigated.

## 5. Conclusions

We have reported innovative results concerning REST, a widely investigated transcription factor known to repress the expression of genes via the generation of histone H3 marks highly active on the epigenomics of multiple DNA regions. The strategy of our study included a few aspects of interest. First, the use of a unique cellular model composed of two clones of PC12 cells: the wtPC12, with very low levels of REST, a property common to mature neurons and many neural cells, and the hrPC12, spontaneously expressing very high levels of the repressor. This model, which does not exhibit the limitations of other neural and nonneural cell models, offers clear advantages, including the fact that, in the future, it could be extended to cells of either clone in which changes of the REST levels had been induced/repressed.

Another aspect of interest was the integration of the RNA-Seq data with two functional approaches, the enrichments from ChIP-Seq datasets and the analyses from the Roadmap Epigenomics databases. This integration, planned also to take care of the context dependence of REST action in different cell types, has been successful by revealing various mechanistic properties of the down- and upregulations of gene expression. Most of the previous studies in this field had been focused on the direct action of the repressor, with little attention for indirect mechanisms. In the present Roadmap Epigenomics databases analyses, the possible direct REST mechanism appeared to account for ~56.0% of the downregulated genes and to be almost inappreciable for the upregulated genes. Moreover, results have identified the possible involvement of PRCs, apparently operative as synergists of REST in the induction of downregulation. On the other hand, two stimulatory TFs Smad3 and Myod1 were found to increase the expression of their target genes. These are among the first data about the upregulation of genes in high-REST neural cells.

We expect the findings of the present study to be useful, in particular for the investigation of the REST epigenomics. In a recent comprehensive presentation REST has been recognized among the TFs that govern the histone marks, most important for multiple genomic regions [32]. The complexity and variability of the gene repression by REST had been demonstrated [48–51]. So far however no favorable models had been available for these studies, and numerous problems remain therefore to be solved.

## Supplementary Material

Short description of Supplementary Materials The supplementary materials report details about the potential REST target genes revealed by RNA-Seq (Si Table 1) and about the Enrichment Analyses of various, specific populations of potential target genes, down- and upregulated in hrREST cells. Specifically, Si Tables 2 and 3 report about ENCODE TF and ChAE genes down- and upregulated; Si Tables 4 and 5 about the pathway enrichment of the same genes; Si Table 6 about TF protein-protein interactions.
Table S1. Differential Expression Analysis by DESEq of hrPC12 down-regulated (log2 Fold change <2) and up-regulated (log2 Fold change >2) genes.
Table S2. ENCODE Transcription Factor (TF) and ChEA database ChIP-seq Enrichment Analysis of down-regulated genes in hrPC12 cells.
Table S3. ENCODE Transcription Factor (TF) and ChEA database ChIP-seq Enrichment Analysis of up-regulated genes in hrPC12 cells.
Table S4. Pathway Enrichment Analysis of down-regulated REST potential targets.
Table S5. Pathway Enrichment Analysis of up-regulated REST potential targets.
Table S6. Transcription Factor Protein-Protein Interaction Network Enrichment Analysis of up-regulated genes.

## Figures and Tables

**Table 1:** ENCODE transcription factor (TF) and ChEA database ChIP-seq enrichment analysis using genes downregulated in hrPC12 cells.

Database	Term	*p* value	*Z*-score	Combined_score	Adj. *p* value
ChEA	REST-18959480-MESC-mouse	3.10*E* − 69	−2.202	338	1.36*E* − 66
ENCODE_TF_ChIP-seq_2015	EZH2_B cell_hg19	8.39*E* − 68	−1.631	241	2.94*E* − 65
ChEA	SUZ12-18692474-MESC-mouse	2.67*E* − 64	−1.587	226	6.66*E* − 62
ChEA	RNF2-18974828-MESC-MOUSE	2.04*E* − 53	−1.81	213	3.24*E* − 51
ChEA	EZH2-18974828-MESC-MOUSE	2.04*E* − 53	−1.798	212	3.24*E* − 51
ChEA	SUZ12-18974828-MESC-MOUSE	9.08*E* − 60	−1.507	199	1.77*E* − 57
ChEA	NR3C1-23031785-PC12-MOUSE	6.47*E* − 42	−2.015	185	5.96*E* − 40
ENCODE_TF_ChIP-seq_2015	REST_HCT116_hg19	3.27*E* − 46	−1.812	179	4.09*E* − 44
ENCODE_TF_ChIP-seq_2015	REST_U-87 MG_hg19	1.79*E* − 45	−1.806	176	1.96*E* − 43
ENCODE_TF_ChIP-seq_2015	REST_Panc1_hg19	2.98*E* − 44	−1.813	172	2.90*E* − 42

Top 10 significant databases (adj. *p* value (Benjamini and Hochberg) < 0.05 and *Z*-score < −1.5) ordered by combined score (−log⁡*p* value*∗Z*-score). For ChEA databases a PMID PubMed id is given after the TF. hg19 stays for human genome GRCh37.

**Table 2:** ENCODE transcription factor (TF) and ChEA database ChIP-seq enrichment analysis using upregulated genes in hrPC12 cells.

Database	Term	*p* value	*Z*-score	Combined_score	Adj. *p* value
ChEA	SOX2-20726797-SW620-HUMAN	5.61*E* − 53	−1.589	183	4.88*E* − 50
ChEA	ZNF217-24962896-MCF7-HUMAN	8.88*E* − 38	−1.507	124	1.19*E* − 35
ChEA	SMAD3-18955504-HaCaT-human	2.48*E* − 37	−1.508	123	2.69*E* − 35
ChEA	ESR1-22446102-UTERI-MOUSE	2.20*E* − 36	−2.178	173	2.01*E* − 34
ChEA	TP53-18474530-U2OS-human	1.74*E* − 28	−1.657	102	8.65*E* − 27
ChEA	EP300-20729851-FORBRAIN_MIDBRAIN_LIMB_HEART-MOUSE	9.48*E* − 25	−1.824	97	3.83*E* − 23
ENCODE_TF_ChIP-seq_2015	TCF12_myocyte_mm9	4.27*E* − 24	−1.84	87	1.68*E* − 22
ENCODE_TF_ChIP-seq_2015	MYOD1_C2C12_mm9	2.64*E* − 20	−1.833	72	8.62*E* − 19
ENCODE_TF_ChIP-seq_2015	MYOD1_myocyte_mm9	5.12*E* − 20	−1.587	62	1.59*E* − 18
ChEA	SOX2-18358816-MESC-mouse	2.64*E* − 19	−1.617	66	8.05*E* − 18

Top 10 significant databases (adj. *p* value (Benjamini and Hochberg) < 0.05 and *Z*-score < −1.5) ordered by combined score (−log⁡*p* value*∗Z*-score). For ChEA databases a PMID PubMed id is given after the TF. mm9 stays for Mus musculus genome assembly NCBI Build 37.
